# Association between Age at Menarche and Hyperhomocysteinemia among Women in Hunan, China: A Cross-Sectional Study

**DOI:** 10.31083/j.rcm2307221

**Published:** 2022-06-24

**Authors:** Yi Yang, Shihong Du, Rong Xie, Yannan Zhang, Tong Yu, Zihao Ding, Xiuqin Hong

**Affiliations:** ^1^First Affiliated Hospital of Hunan Normal University, Hunan Provincial People’s Hospital, 410000 Changsha, Hunan, China; ^2^Key Laboratory of Molecular Epidemiology of Hunan Province, 410000 Changsha, Hunan, China

**Keywords:** age at menarche, hyperhomocysteinemia, hypertension, cardiovascular disease

## Abstract

**Background:**

We aim to examine the relationship between age at menarche and hyperhomocysteinemia in women in Hunan Province.

**Methods:**

Participants were required to complete a questionnaire that included age at menarche, lifestyle habits, other baseline information, and blood biochemical parameters in a cross-sectional study. The association between hyperhomocysteinemia and age at menarche was examined by Multivariable adjusted logistic regression.

**Results:**

A cohort of 2008 women with a mean age of 60.11 years (aged from 18.0 to 88.0 years) was included in this study. After adjustment for confounding factors such as age, the results showed that the risk of hyperhomocysteinemia among women whose age at menarche were over 16 years was 2.543 (1.849, 3.469) times higher than the risk among women whose age at atmenarche were less than 14 years, and 2.656 (1.882, 3.748) times more likely to have hypertension than women with menarche at 14 years. Besides, the odds ratios of diabetes, hyperlipidemia, and obesity were elevated in women older than 16 years of age at menarche (OR = 1.924, *p *< 0.001; OR = 1.491, *p* = 0.014; OR = 1.670, *p* = 0.022).

**Conclusions:**

Our findings suggest that late menarche tends to be associated with a high risk of hyperhomocysteinemia and its associated set of diseases such as hypertension, diabetes, hyperlipidemia, and obesity in women in Hunan, China. This association tends to differ across birth cohorts. Therefore, adequate attention of menarcheal age may be able to predict diseases in elderly females.

## 1. Introduction

Hyperhomocysteinemia (HHcy) has been defined as an elevated serum concentration of homocysteine exceeding 15 μmol/L [1]. In 2018, the prevalence of HHcy among Chinese adults was 37.2% [2], and has increased year by year. Current evidence suggests that the HHcy is closely associated with the onset and development of vascular endothelial injury [3] and elevated risk of cardiovascular disease (CVD) [4]. HHcy has become a worldwide public health problem. Earlier studies have suggested that menarche is associated with the development and mortality of CVD events [5, 6]. However, previous studies have focused on Western women born before the 1960s and have reached inconsistent conclusions. In addition to the prevailing view that early menarche leads to disease [5], some studies have concluded that menarche is not associated with cardiovascular disease [7]. In contrast, some have found a U-shaped association between age at menarche and vascular-related disease [8]. However, data from Chinese women show that Chinese women born in the same era tend to have a later age at menarche than European populations [9]. Analysis of data from various European countries shows that the age at menarche is 12.5 years in those who born before the 1950s [10]. However, Chinese women born before the 1950s had an age at menarche of 16 years [11]. The relationship between age at menarche and HHcy has not been previously studied. We wondered whether age at menarche takes a predicting role in the development of HHcy when Chinese women are generally older age at menarche.

## 2. Materials and Methods

### 2.1 Participants

The participants of our study were selected from four departments (Cardiology, neurology, geriatrics, general practice) of Hunan Provincial People’s Hospital between 2018–2021. After excluding participants who were male, taking vitamin B6, vitamin B12, folic acid, using sex hormones (estrogen or progesterone) and other medications that affect estrogen, Hcy levels, lipid, cardiovascular function, blood pressure, fail to respond and age at menarche earlier than 8 years (n = 0) or later than 22 years (n = 4), 2008 female inpatients aged 18–88 were selected. All patients participating in the study were informed and consented to this study.

### 2.2 Data Collection

Information on baseline characteristics of participants (age, gender, registration address, marital status, etc.) lifestyles (smoking status, drinking status) was obtained. Patients were instructed to complete the questionnaire face-to-face and one-to-one by well-trained investigators. Smoking at least one cigarette a day for six months was defined as smoking, and drinking alcohol at least 12 times per year was defined as alcohol consumption. Body mass index (BMI, kg/$m^{2}$) was calculated as weight (kg) divided by height (m) squared. Venous blood and urine samples were collected from all participants after fasting for at least eight hours, and all samples were sent to the laboratory department of Hunan Provincial People’s Hospital within 4 hours. E2 (estradiol) was measured by electro-chemiluminescence immunoassay, while Hcy (homocysteine) was measured by the enzymatic cycling method. The other general demographic information (place of residence, whether menopause, age at menopause, etc.) and biochemical indicators, such as GFR (glomerular filtration rate), LDL-C (low-density lipoprotein cholesterol), TC (total cholesterol), TG (triglyceride), UA (uric acid), ALT (alanine aminotransferase), BUN (blood urea nitrogen), DBP (diastolic blood pressure), FPG (fasting plasma glucose), HDL-C (high-density lipoprotein cholesterol), SBP (systolic blood pressure), Scr (serum creatinine), were collected following standard procedure for laboratory department of Hunan Provincial People’s Hospital.

### 2.3 Definitions

Hypertension was defined as systolic blood pressure ≥140 mmHg and/or diastolic blood pressure ≥90 mmHg [12]. Diabetes was defined as FPG ≥7.0 mmol/L [13]. Hyperlipidemia was defined as TC ≥6.2 mmol/L and/or TG ≥2.3 mmol/L [14].

### 2.4 Statistical Analysis

The data were double-inputted with EpiData 3.1 (EpiData Association, The Kingdom of Denmark), and SPSS 26.0 (IBM, Armonk, NY, USA) software package was used to perform statistical analysis. We divided participants into five groups based on the plural and quartiles of their age at menarche: ≤12, 13, 14, 15, and ≥16. In addition, because of the possible influence of birth year on age at menarche, we divided the birth cohort into <1950, 1950–1970, and >1970 based on particular events in the Chinese historical process. Age at menarche was modeled as a linear variable while HHcy was modeled as categorical variables.

The categorical variables are presented as percentages and frequencies, while the continuous variables are described as mean ± standard deviation. Differences in baseline information between the five groups were assessed by Analysis of Variance (continuous variables) and chi-square tests (categorical variables). To explore the associations between age at menarche and blood biochemical indices, a linear regression model was developed. No adjustment for other factors (model 1), adjustment for age at entry (model 2), and adjustment for other confounding factors such as age, marital status, and age at menopause (model 3), respectively. As stated, we further investigated the association between age at menarche and diseases (HHcy, hypertension, diabetes, Hyperlipidemia, and obesity) using a multivariate-adjusted regression model. To assess whether this association was related to the birth cohort, we stratified the sample by birth year to examine the association between age at menarche and diseases (HHcy and hypertension) at different birth years. Results were presented as odds ratios (ORs) with 95% CIs. In all logistic regressions, age at menarche equal to 14 was chosen as the reference group because it represents a concentrated trend in the sample. Ultimately, *p* values less than 0.05 were considered statistically significant.

## 3. Results

The basic characteristics of the study participants stratified by age at menarche are summarized in Table 1. We included 2008 study subjects, whose mean age was 60.11 ± 11.60 years (aged from 18.0 to 88.0 years). The mean age at menarche was 14.06 ± 1.41 years (aged from 10.0 to 20.0 years). Women with a later menarcheal age were more likely to be older, be in menopause, and have children earlier. Later age at menarche was accompanied by higher SBP, HCY, FPG, UA, BUN, TC, E2, and lower GFR than women with earlier menarche.

**Table 1.** **Characteristics of the participants according to age at menarche**.

Characteristics	Total	Age at menarche, y					*p*
		≤12	13	14	15	≥16	
N	2008	180	560	700	314	254	——
Age at enrollment, y	60.11 ± 11.60	58.54 ± 12.04	57.74 ± 10.92	59.17 ± 11.67	63.38 ± 11.24	65.00 ± 11.60	<0.001
Urban, n (%)	1170 (58.3)	103 (57.2)	351 (56.3)	400 (57.1)	197 (62.7)	155 (61.0)	0.329
Menopause, n (%)	1708 (85.1)	148 (82.2)	460 (82.1)	580 (82.9)	284 (90.4)	236 (92.9)	<0.001
Age at menopause, y	49.62 ± 3.25	49.66 ± 3.18	49.76 ± 2.96	49.53 ± 3.46	49.53 ± 3.30	49.65 ± 3.25	0.818
Menstruation is regular, n (%)	1861 (96.3)	168 (93.3)	512 (91.4)	652 (93.1)	300 (95.5)	229 (90.2)	0.004
Age at first delivery, y	24.01 ± 3.17	24.39 ± 3.15	23.88 ± 3.12	24.10 ± 3.11	24.33 ± 3.50	23.41 ± 2.93	0.003
Age at last delivery, y	27.14 ± 3.85	27.25 ± 3.78	26.72 ± 3.70	27.22 ± 3.92	27.51 ± 3.94	27.15 ± 3.84	0.102
BMI	23.69 ± 5.39	24.14 ± 9.72	23.35 ± 3.49	23.81 ± 6.28	23.46 ± 3.31	24.05 ± 3.68	0.248
SBP, mm Hg	135.52 ± 21.63	136.52 ± 22.66	133.58 ± 20.82	134.29 ± 21.29	139.19 ± 22.85	137.94 ± 21.36	0.001
DBP, mm Hg	80.80 ± 28.46	81.10 ± 13.58	79.89 ± 12.03	81.63 ± 45.18	80.57 ± 11.73	80.62 ± 12.71	0.877
Hcy, μmol/L	13.19 ± 5.87	13.41 ± 6.82	12.68 ± 5.98	12.71 ± 6.42	13.83 ± 4.37	14.66 ± 4.52	<0.001
FPG, mmol/L	5.66 ± 2.34	5.84 ± 2.60	5.38 ± 1.93	5.56 ± 2.24	5.58 ± 2.08	6.49 ± 3.15	<0.001
BUN, mmol/L	65.82 ± 123.85	67.60 ± 131.36	90.18 ± 138.17	69.91 ± 127.23	44.76 ± 105.52	25.74 ± 72.84	<0.001
UA, μmol/L	259.75 ± 154.26	255.46 ± 150.93	232.90 ± 166.00	258.24 ± 159.69	280.33 ± 135.85	300.36 ± 121.36	<0.001
GFR, mL/min	90.35 ± 24.62	88.98 ± 28.81	92.80 ± 22.52	91.95 ± 24.80	86.33 ± 23.53	86.13 ± 25.78	0.001
Scr, μmol/L	72.25 ± 79.84	73.79 ± 66.68	71.02 ± 72.41	73.01 ± 98.03	71.18 ± 68.17	73.06 ± 59.28	0.987
ALT, U/L	21.37 ± 19.24	20.14 ± 15.04	21.45 ± 21.28	21.07 ± 18.92	21.64 ± 18.59	22.49 ± 18.90	0.772
TC, mmol/L	4.60 ± 1.18	4.44 ± 1.03	4.59 ± 1.10	4.59 ± 1.25	4.56 ± 1.09	4.82 ± 1.35	0.013
TG, mmol/L	1.82 ± 1.40	1.85 ± 1.21	1.79 ± 1.32	1.81 ± 1.47	1.80 ± 1.16	1.91 ± 1.69	0.802
LDL-C, mmol/L	2.71 ± 0.91	2.56 ± 0.87	2.72 ± 0.89	2.66 ± 0.92	2.72 ± 0.89	2.90 ± 0.95	0.001
HDL-C, mmol/L	1.25 ± 0.35	1.20 ± 0.34	1.27 ± 0.41	1.23 ± 0.34	1.26 ± 0.31	1.28 ± 0.32	0.035
E2, pg/mL	24.26 ± 25.24	29.49 ± 38.08	24.62 ± 23.69	24.06 ± 23.13	23.40 ± 24.35	20.83 ± 23.09	0.049

When the study participants were grouped by birth cohort, it was found that women with an earlier birth date generally had later menarche and had a slightly later age of menopause than women with a later birth cohort. Women who were born earlier also had an earlier age at first delivery and a later age at final delivery. Lifestyle habits such as smoking and alcohol consumption did not differ between the three groups. These data are presented in Table 2.

**Table 2.** **Characteristics of the participants according to birth cohort**.

Characteristics	Birth cohort			*p*
	<1950	1950–1970	>1970	
N	557	1200	251	—
Age at enrollment, y	74.04 ± 5.72	57.56 ± 5.36	41.42 ± 7.04	<0.001
Urban, n (%)	397 (71.3)	658 (54.8)	115 (45.8)	<0.001
Married (n%)	380 (68.2)	1130 (94.3)	229 (91.2)	<0.001
Age at menarche, y	14.50 ± 1.58	13.92 ± 1.33	13.76 ± 1.17	<0.001
Age at menopause, y	49.74 ± 2.90	49.67 ± 3.34	46.00 ± 3.60	<0.001
Age at first delivery, y	23.53 ± 3.52	24.17 ± 3.00	24.44 ± 2.90	<0.001
Age at last delivery, y	28.35 ± 3.95	26.42 ± 3.53	27.00 ± 4.21	<0.001
Smoking, n (%)	12 (2.2)	33 (2.9)	99 (4.0)	0.292
Alcohol, n (%)	19 (3.4)	38 (3.2)	5 (2.2)	0.618

Linear regression analysis showed that UA, TC, LDL-C, Hcy, and FPG were all positively correlated with age at menarche, which suggests that the later age at menarche, the higher risk of disease. These data are presented in Table 3.

**Table 3.** **Relationship between biochemical index and age at menarche**.

Variable	Model 1		Model 2		Model 3	
	β	*p*	β	*p*	β	*p*
UA, μmol/L	14.175	<0.001	6.862	0.004	5.411	0.020
TC, mmol/L	0.068	<0.001	0.068	<0.001	0.075	<0.001
TG, mmol/L	0.037	0.092	0.035	0.121	0.036	0.116
LDL-C, mmol/L	0.057	<0.001	0.056	<0.001	0.060	0.031
HDL-C, mmol/L	0.007	0.185	0.006	0.275	0.008	0.176
Hcy, μmol/L	0.416	<0.001	0.218	0.023	0.208	0.032
FPG, mmol/L	0.154	<0.001	0.105	0.012	0.104	0.014
E2, pg/mL	–1.401	0.003	0.152	0.722	–0.102	0.808

Model 1: we did not adjust any confounding factors. Model 2: we adjusted the age. Model 3: we adjusted age, area of residence, marital status, age at menopause, age at first delivery, age at final delivery, smoking, and alcohol consumption.

Multivariable logistic regression analysis showed that women with age at menarche of 13 years had a lower risk of developing hypertension and diabetes than women with age at 14 years (OR = 0.740, *p* = 0.009; OR = 0.597, *p* = 0.003). Conversely, women with age at menarche older than 16 years had the risk of HHcy 2.543-fold and hypertension 2.656-fold compared with women with age at menarche 14 years. The odds of diabetes, hyperlipidemia, and obesity were elevated in women older than 16 years of age at menarche (OR = 1.924, *p *< 0.001; OR = 1.491, *p* = 0.014; OR = 1.670, *p* = 0.022). This part of the data is shown in Table 4.

**Table 4.** **Adjusted ORs for disease according to the age at menarche**.

Variable	Age at menarche, y								
	≤12		13		14	15		≥16	
	OR	*p*	OR	*p*	OR	OR	*p*	OR	*p*
HHcy	1.164 (0.781, 1.174)	0.455	1.017 (0.770, 1.343)	0.906	1	2.046 (1.511, 2.770)	<0.001	2.543 (1.849, 3.469)	<0.001
Hypertension	1.098 (0.783, 1.540)	0.588	0.740 (0.591, 0.927)	0.009	1	1.842 (1.373, 2.471)	<0.001	2.656 (1.882, 3.748)	<0.001
Diabetes	0.925 (0.582, 1.471)	0.743	0.597 (0.423, 0.844)	0.003	1	1.164 (0.815, 1.662)	0.403	1.924 (1.360, 2.724)	<0.001
Hyperlipidemia	0.755 (0.500, 1.140)	0.181	1.186 (0.918, 1.532)	0.191	1	1.364 (1.012, 1.839)	0.041	1.491 (1.086, 2.047)	0.014
Obesity	1.264 (0.742, 2.152)	0.388	0.820 (0.546, 1.232)	0.339	1	0.725 (0.434, 1.210)	0.218	1.670 (1.078, 2.586)	0.022

The model was corrected for age, area of residence, marital status, menopause, smoking, and alcohol consumption.

After stratifying the study population by birth cohort, women born before 1950 and 1950–970 cohorts had a higher risk of HHcy if they had later menarche (OR = 2.354, *p *< 0.001; OR = 1.829, *p* = 0.016), but women born after 1970 had a higher risk of hypertension if they had a later age at menarche (OR = 2.517, *p* = 0.011). These data are presented in Table 5.

**Table 5.** **Adjusted ORs for HHcy and hypertension associated with age at menarche by birth cohort**.

Variable	birth cohort	Age at menarche, y								
		<12		13		14	15		>16	
		OR	*p*	OR	*p*	OR	OR	*p*	OR	*p*
HHcy	<1950	1.312 (0.634, 2.716)	0.464	1.704 (1.042, 2.785)	0.034	1	2.349 (1.432, 3.853)	0.001	2.354 (1.445, 3.835)	0.001
	1950–1970	1.357 (0.797, 2.311)	0.261	0.891 (0.603, 2.318)	0.564	1	1.671 (1.070, 2.609)	0.024	1.829 (1.120, 2.987)	0.016
	>1970	0.558 (0.116, 2.676)	0.466	0.788 (0.292, 2.127)	0.638	1	0.802 (0.209, 3.080)	0.748	1.283 (0.250, 6.588)	0.765
Hypertension	<1950	1.023 (0.326, 3.210)	0.969	1.073 (0.487, 2.367)	0.861	1	1.607 (0.675, 3.829)	0.284	2.595 (0.935, 7.199)	0.067
	1950–1970	1.071 (0.706, 1.626)	0.746	0.727 (0.550, 0.962)	0.026	1	1.405 (0.970, 3.036)	0.072	1.763 (1,146, 2.714)	0.010
	>1970	1.846 (0.759, 4.491)	0.177	0.702 (0.352, 1.397)	0.313	1	2.349 (1.008, 5.474)	0.048	2.517 (1.132, 7.341)	0.011

## 4. Discussion

The results of this study showed that compared to those who were born before 1950, women born after the 1970s had an earlier age at menarche from 14 to 13 years, and this decrease in the age at menarche was not exceptional. In a study based on data from the China Health and Nutrition Survey, the average age at menarche for Chinese women dropped dramatically from 14.35 to 12.60 years from 1973 to 2004 [15]. The decline in the age at menarche among women in developed countries has been slightly slower; for example, the average age at menarche among Japanese women of the same Asian ethnicity decreased from 13.8 to 12.2 years, a decline of 1.6 years over approximately 50 years [16]. The average age at menarche dropped from 12.75 to 12.54 years Over 25 years in the United States [17]. However, the current study shows that the decline in the age at menarche in Chinese women has not slowed down.

Previous studies have suggested that earlier age at menarche may lead to increased cardiovascular disease and mortality [18], including hypertension and poor glucose tolerance [19]. Earlier age at menarche leads to increased plasma cholesterol or adverse vascular changes in postmenopausal women [11]. In 2016, a study of the association between age at menarche and cardiovascular disease among 300,000 women in 10 regions of China found that this association varied between birth cohorts [20]. Women who are born in the early cohort usually had a later age at menarche. However, the present study found women generally have an earlier age at menarche, while a later age at menarche may be associated with a range of disorders in middle-aged and elderly women. The pattern of reproductive factors and their interaction with lifestyle factors may explain the increased risk of cardiovascular disease. The industrialization revolution in China in the 1970s allowed young women to receive better education, more time for exercise, a later age at first birth, fewer births, and less smoking and alcohol use than women born in the 1950s.

The results of this study showed that advancing within normal limits, an earlier age at menarche is protective against diabetes and hypertension (OR = 0.740, *p* = 0.009; OR = 0.597, *p* = 0.003). However, this protective effect disappears when the age at menarche is less than 12 years. Most studies from the West, Japan, and developed cities on the eastern coast of China have shown that early menarche increases the risk of hypertension. Still, our study demonstrated that late age at menarche is associated with an increased risk of hypertension (OR = 2.656, *p *< 0.001). Such results may be related to the fact that the Hunan region was economically underdeveloped in the last century and that the lifestyle and reproductive factors of the study subjects may influence the disease in middle and old age. A study that counted women born during the Great Depression in the United States showed that both early or late age at menarche contributed to the development of diabetes [21]. Similarly, late age at menarche was only found to be associated with a high risk of hypertension in the economically underdeveloped southwest region of China [22]. Few previous studies have taken into account differences across birth years and living conditions. We suggest that age at menarche affects women with different diseases in middle and old age under various economic conditions and different generations, or, in other words, under different lifestyle influences.

It has been well established that early menarche leads to obesity in adolescents and young women [23], but very few studies have been conducted on postmenopausal women because confounding factors of age cannot be excluded. In the present study, after excluding the effects of age and reproductive factors, late menarche was still associated with obesity (OR = 1.670, *p* = 0.022). Furthermore, age at menarche was associated with a stronger relationship of cardiovascular disease in white women compared to black people, both in adolescents [24] and in mature women [21]. Considering that age at menarche also differs between Chinese and European women, we speculate whether there are also ethnic differences in age at menarche, and, at the same time, the association between age at menarche and disease.

As an independent risk factor for CVD, Homocysteine is of great importance for the prediction of CVD. For women in Asia, the health consequences of reproductive factors on CVD risks have not been well quantified. If possible, risk factors for HHcy can be identified and controlled early in the life course, and it will have significant implications for health in women’s middle and old age. Indeed, besides cardiovascular disease, HHcy has been suggested as a mediator of other diseases. Due to the adverse vascular effects and cytotoxicity of homocysteine, HHcy can affect almost all body systems. Such as dementia and Alzheimer’s disease [25], osteoporosis [26], diabetes mellitus [27], and diabetic retinopathy [28]. Therefore the relationship between age at menarche and HHcy needs to be urgently studied.

The underlying mechanism for the difference in the age at menarche is unclear. A genome-wide association study in European women identified 106 genomic loci associated with age at menarche [29, 30], but it is unknown whether this evidence can be applied to Asian women. The decline in endogenous estrogen in postmenopausal women is associated with the risk of cardiovascular disease [31]. In women born earlier than the 1950s, age at menarche remained negatively associated with estrogen after age correction (β = –1.714, *p* = 0.008) [22]. At the same time, our findings showed that estrogen was lower at the later age at menarche in the whole population (β = –1.401, *p* = 0.003). Although the confounder of age cannot be ruled out, considering the covariance between age and age at menarche, we still consider this result as a guideline. Therefore, it is reasonable to speculate whether the effect of age at menarche on cardiovascular disease is mediated by estrogen. However, it has been suggested that some of the effects of earlier age at menarche on metabolic disease may be mediated through adult BMI levels [32]. In the present study, we similarly found that later age at menarche may be associated with an increased risk of obesity (OR = 1.670, *p* = 0.022). Also, we included the BMI-defined obesity variable, corrected for the effect of obesity on Hcy levels in middle-aged and elderly women, and our analysis revealed that age at menarche could affect plasma Hcy concentrations independently of the obesity variable. This implies that there may be a biological mechanism by which age at menarche affects plasma Hcy concentrations in adult women independently of obesity.

## 5. Limitations

Our study has several limitations. First, our sample spanned a large age range (18–88 years), and although stratified analyses by corrected age and birth cohort were able to offset confounding factors to some extent, most participants were older, with a smaller number of participants born after 1970. Second, when baseline information on age at menarche was collected, there may have been recalling bias in the data for some participants due to age. Finally, the samples collected in this study were all from inpatients of Hunan Provincial People’s Hospital, which is located in Changsha, the capital of Hunan Province, and most of the samples collected were from Changsha and its surrounding cities, which may have caused the existence of some selection bias.

## 6. Conclusions

Our findings suggest that late menarche tends to be associated with a high risk of hyperhomocysteinemia and its associated set of diseases such as hypertension, diabetes, hyperlipidemia, and obesity in women in Hunan, China. This association tends to differ across birth cohorts. Therefore, adequate attention to menarcheal age may be able to predict diseases in older women.
